# Computed tomography coronary angiography in non-ST-segment elevation myocardial infarction

**DOI:** 10.1259/bjr.20220346

**Published:** 2022-09-19

**Authors:** Kang-Ling Wang, Mohammed N Meah, Anda Bularga, Trisha Singh, Michelle C Williams, David E Newby

**Affiliations:** ^1^ Centre for Cardiovascular Science, University of Edinburgh, Edinburgh, United Kingdom; ^2^ General Clinical Research Center, Taipei Veterans General Hospital, Taipei, Taiwan; ^3^ School of Medicine, National Yang Ming Chiao Tung University, Taipei, Taiwan

## Abstract

Electrocardiography and high-sensitivity cardiac troponin testing are routinely applied as the initial step for clinical evaluation of patients with suspected non-ST-segment elevation myocardial infarction. Once diagnosed, patients with non-ST-segment elevation myocardial infarction are commenced on antithrombotic and secondary preventative therapies before undergoing invasive coronary angiography to determine the strategy of coronary revascularisation. However, this clinical pathway is imperfect and can lead to challenges in the diagnosis, management, and clinical outcomes of these patients. Computed tomography coronary angiography (CTCA) has increasingly been utilised in the setting of patients with suspected non-ST-segment elevation myocardial infarction, where it has an important role in avoiding unnecessary invasive coronary angiography and reducing downstream non-invasive functional testing for myocardial ischaemia. CTCA is an excellent gatekeeper for the cardiac catheterisation laboratory. In addition, CTCA provides complementary information for patients with myocardial infarction in the absence of obstructive coronary artery disease and highlights alternative or incidental diagnoses for those with cardiac troponin elevation. However, the routine application of CTCA has yet to demonstrate an impact on subsequent major adverse cardiovascular events. There are several ongoing studies evaluating CTCA and its associated technologies that will define and potentially expand its application in patients with suspected or diagnosed non-ST-segment elevation myocardial infarction. We here review the current evidence relating to the clinical application of CTCA in patients with non-ST-segment elevation myocardial infarction and highlight the areas where CTCA is likely to have an increasing important role and impact for our patients.

## Introduction

Acute chest pain is one of the commonest presentations to the Emergency Department, with half of the patients subsequently being hospitalised for assessment of suspected acute coronary syndrome.^1^ It accounts for 6% of all Emergency Department attendances in the UK,^2^ resulting in approximately 350,000 hospitalisations each year. In contemporary practice, the application of high-sensitivity cardiac troponin assays for the diagnosis of myocardial infarction is central to clinical pathways for acute chest pain.^3–5^ This has resulted in marked improvements in the detection of not only myocardial infarction but also myocardial injury, substantially altering the prevalence of the diagnoses of unstable angina and myocardial infarction.^6,7^ Moreover, high-sensitivity cardiac troponin assays can expedite the exclusion of myocardial infarction through so-called ‘rule-out’ pathways.^8^ Indeed, less than one in five patients are ultimately diagnosed with an acute coronary syndrome.^9,10^ However, because of their high sensitivity, these assays also have issues of specificity, and a large proportion of cardiac troponin elevations are unrelated to atherosclerotic plaque disruption or even not myocardial infarction.^11^

The differentiation between the various mechanisms responsible for myocardial injury and distinctive subtypes of myocardial infarction is critical because those with a classic or Type 1 myocardial infarction attributable to atherosclerotic plaque disruption are more likely to benefit from intensive pharmacotherapy and coronary revascularisation. Interestingly, only a half or less of patients admitted with cardiac troponin elevation are diagnosed with Type 1 myocardial infarction.^12,13^ Furthermore, neither cardiac troponin concentration thresholds nor background cardiovascular risk factors are sufficient to identify underlying mechanisms of myocardial injury nor determine the subtypes of myocardial infarction.^14,15^ Therefore, invasive coronary angiography plays a pivotal role in the management of high-risk patients with acute chest pain,^5^ including those with cardiac troponin elevation.

Computed tomography coronary angiography (CTCA) has diagnostic accuracy and prognostic performance comparable to invasive coronary angiography in the diagnosis of obstructive coronary artery disease. It is associated with enhancing clinical diagnosis, better targeting of treatments, and improving clinical outcomes in patients with stable chest pain.^16,17^ Moreover, CTCA is cost-effective and reduces length of stay in patients with acute chest pain and without cardiac troponin elevation.^18,19^ To exclude underlying obstructive coronary artery disease, the European Society of Cardiology guidelines recommend CTCA for those with chronic coronary syndrome and a lower likelihood of the presence of haemodynamically significant stenosis,^20^ and for those with suspected acute coronary syndrome and a normal or inconclusive cardiac troponin concentration.^21^ However, it remains unclear whether patients with non-ST-segment elevation myocardial infarction would also benefit from CTCA by improving their downstream management and treatment. This review will examine the latest advances in CTCA in patients with acute chest pain, discuss the potential application of CTCA in those with suspected non-ST-segment elevation myocardial infarction, and highlight potential future developments in this area.

## Diagnosis of non-ST-segment elevation myocardial infarction

Any elevation of a cardiac troponin concentration above the 99th centile upper reference limit defines myocardial injury. Under such a pre-requisite, myocardial infarction is diagnosed by a rise and fall in cardiac troponin in the context of clinical symptoms or electrocardiographic signs suggestive of myocardial ischaemia. Based on underlying pathophysiology contributing to oxygen supply–demand imbalance, myocardial infarction is further subclassified into five types: atherothrombosis (Type 1), acute mismatch between oxygen supply and demand unrelated to atherosclerotic plaque disruption (Type 2), cardiac death presumably due to myocardial ischaemia (Type 3), and consequences of coronary procedures (Types 4 and 5).^22^ Finally, an elevation in cardiac troponin in the absence of symptoms or signs of ischaemia is termed myocardial injury and can be acute (rise and fall of cardiac troponin) or chronic (persistently elevated). Although non-ischaemic aetiologies account for the majority of cardiac troponin elevations,^23^ differentiating and classifying myocardial injury and myocardial infarction are not always straightforward even among a panel of experts with pre-specified guidance.^24,25^

The presence of coronary atherosclerosis is a pre-requisite for Type 1 myocardial infarction and a common observation in Type 2 myocardial infarction. Although assessing coronary artery anatomy may aid in the distinction between myocardial injury and myocardial infarction, invasive coronary angiography has many limitations, especially when the infarct size is small.^26,27^ Currently, cardiac magnetic resonance imaging is recommended in patients with cardiac troponin elevation of undetermined aetiology.^28^ Based on the pattern of injury and dysfunction,^29^ it may distinguish myocardial infarction from myocardial injury,^30^ or alter a location of the infarct-related artery in non-ST-segment elevation myocardial infarction.^31^ However, the pattern of myocardial injury on imaging is not always specific for a disease or pathophysiological process,^32^ and the availability of cardiac magnetic resonance imaging service is limited.^33^

**Figure 1.**
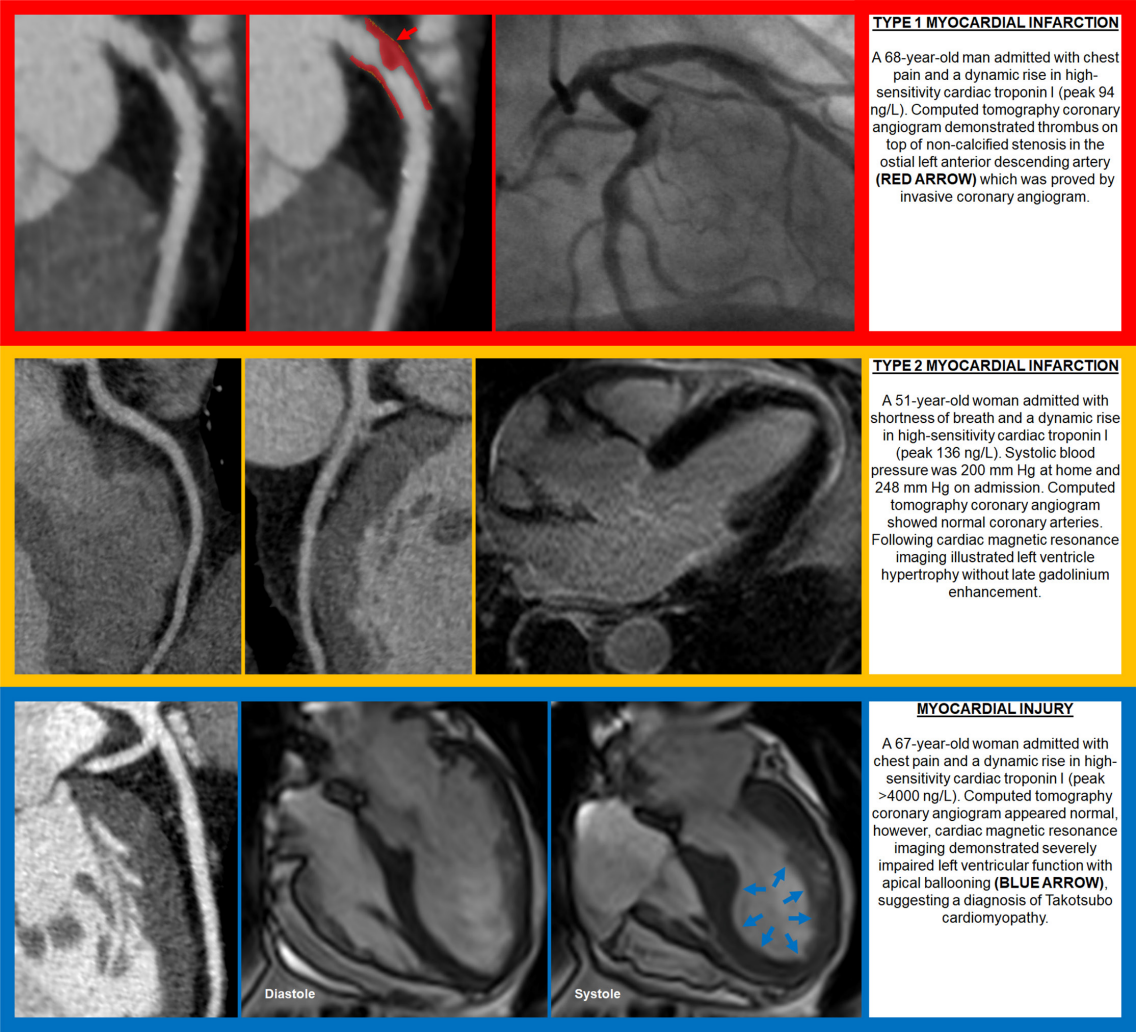
Examples showing the use of computed tomography coronary angiography in differentiation between aetiologies for cardiac troponin elevation

CTCA gives ready access to the non-invasive evaluation of coronary artery anatomy and any associated coronary artery disease, potentially avoiding the need for invasive coronary angiography.^34^ In those with cardiac troponin elevation in the absence of obstructive coronary artery disease, CTCA has greater sensitivity for the detection of atherosclerotic plaques and the identification of the infarct-related artery than invasive coronary angiography.^35^ Conversely, CTCA demonstrates that three-quarters of patients with cardiac troponin elevation and low clinical suspicion for acute coronary syndrome have no or minimal atherosclerotic plaques.^36^ Consequently, the likelihood of Type 1 myocardial infarction is extremely low when all coronary arteries are free from atherosclerotic plaques. Finally, it also offers an opportunity to evaluate other cardiothoracic structures and recognise alternative causes of myocardial injury. Therefore, CTCA provides important information to help guide the diagnosis and classification of aetiologies for myocardial injury (Figure 1).

CTCA has excellent diagnostic performance compared to invasive coronary angiography in patients with stable chest pain and a low-to-intermediate pre-test probability of coronary artery diameter stenosis ≥50%.^37^ Furthermore, the diagnostic performance of CTCA in patients with non-ST-segment elevation myocardial infarction has been demonstrated to be robust in the sub-study of the Very Early Versus Deferred Invasive Evaluation Using Computerized Tomography (VERDICT) trial.^38^ In this trial, 1023 out of 2147 patients with non-ST-segment elevation acute coronary syndrome (78% with myocardial infarction) underwent CTCA before invasive coronary angiography. Using invasive coronary angiography as the gold standard, the negative-predictive value for CTCA to exclude any diameter stenosis ≥50% was 91%. Among 24 patients who were falsely excluded by CTCA, only three had a lesion with diameter stenosis ≥50% in a major epicardial coronary artery by invasive coronary angiography. The positive-predictive value for CTCA to identify diameter stenosis ≥50% was 88%. Among 92 patients who were falsely included by CTCA, 17 had a non-diagnostic scan and 19 had previous coronary stenting. The negative-predictive value for patients with cardiac troponin elevation, an ischaemic electrocardiographic change, and a GRACE score >140 was 89%, 95%, and 95%, respectively. The overall negative- and positive-predictive values of CTCA remained similar when considering diameter stenosis ≥70% as the cut-off. Thus, CTCA has very similar diagnostic performance to invasive coronary angiography in the setting of non-ST-segment elevation myocardial infarction. It has excellent negative-predictive value and rarely misses an obstructive atherosclerotic lesion in a major epicardial coronary artery.

## Management of non-ST-segment elevation myocardial infarction

Non-ST-segment elevation myocardial infarction represents three-quarters of all contemporary myocardial infarctions, but many do not have obstructive coronary artery disease at the time of invasive coronary angiography.^39–41^

Prior to the advent of high-sensitivity cardiac troponin testing, randomised controlled trials of low-risk patients with acute chest pain reported that rates of invasive coronary angiography and coronary revascularisation within usual clinical care were low at less than 6 and 3%, respectively (Table 1).^42^ Implementation of CTCA was associated with a slight increase in the immediate use of invasive coronary angiography. However, around 50% of patients were directly discharged from the Emergency Department after CTCA, and fewer patients required subsequent non-invasive functional testing for myocardial ischaemia,^18,19^ resulting in shorter lengths of stay and a reduced cost at the Emergency Department. Similarly, compared with routine functional ischaemia testing, CTCA was associated with a higher rate of invasive coronary angiography and subsequent coronary revascularisation but again shorter lengths of stay.^43–45^

**Table 1.** Summary of clinical management in major randomised controlled trials in suspected or diagnosed non-ST-segment elevation myocardial infarction

	Patients with low risk^*a*^					Patients with intermediate risk^*b*^		
	ACRIN PA 4005	ROMICAT II	CATCH	CT-COMPARE	CAD-Man	BEACON	RAPID-CTCA	CARMENTA
Controlled treatment	SoC by clinicians' discretion	SoC by clinicians' discretion	Exercise ECG or MPI	Exercise ECG	Invasive coronary angiography	SoC by clinicians' discretion	SoC by clinicians' discretion	SoC by clinicians' discretion
Invasive coronary angiography	5% *vs* 4%	12% *vs* 8%	17% *vs* 12%	7% *vs* 3%	14% *vs* 100%	17% *vs* 13%	54% *vs* 61%	70% *vs* 100%
Diagnostic yield of obstructive CAD among patients referred to invasive coronary angiography	76% *vs* 44%	NR	71% *vs* 36%	92% *vs* 44%	75% *vs* 15%	NR	NR	85% *vs* 61%
Overall coronary revascularisation	3% *vs* 1%	5% *vs* 3%	10% *vs* 4%	4% *vs* 1%	10% *vs* 14%	9% *vs* 7%	34% *vs* 33%	NR
Length of stay	18 hours *vs* 25 hours	23 hours *vs* 31 hours	NR	14 hours *vs* 20 hours	30 hours *vs* 53 hours	6 hours *vs* 6 hours	2.2 days *vs* 2.0 days	4 days *vs* 5 days
Direct discharge	50% *vs* 23%	47% *vs* 12%	NR	NR	NR	65% *vs* 59%	NR	NR
Cost of care at 1 month	NR	$4289 *vs* $4060	NR	$2193 *vs* $2704	NR	€337 *vs* €511	NR	NR
Downstream non-invasive ischaemia testing	NR	20% *vs* 80%	2% *vs* 3%	NR	NR	4% *vs* 11%	19% *vs* 26%	NR
Additional finding	NA	NA	NA	NA	CTCA was associated with a lower rate of minor procedural complications (4% *vs* 11%)	NA	CTCA did not alter the rate of subsequent change in preventive treatments (63% *vs* 62%)	NA

CAD, coronary artery disease; CTCA, computed tomography coronary angiography; ECG, electrocardiogram; MPI, myocardial perfusion imaging; NA, not applicable; NR, not reported; SoC, standard of care.

The RAPID-CTCA trial reported the proportion of patients having abnormal ECG.

Statistics compare between CTCA and the controlled treatments.

a Patients had neither history of CAD, ischaemic ECG, nor cardiac troponin elevation.

b Patients had either history of CAD, ischaemic ECG, or high-sensitivity cardiac troponin elevation.

In the era of high-sensitivity cardiac troponin testing, three randomised controlled trials have evaluated the clinical effectiveness of CTCA in patients presenting with suspected or diagnosed non-ST-segment elevation myocardial infarction (Table 1). The Better Evaluation of Acute Chest Pain with Computed Tomography Angiography (BEACON) trial recruited 500 patients with suspected acute coronary syndrome with only a minority (5%) having modest cardiac troponin elevation (within three times 99th centile upper reference limit). In contrast, the Rapid Assessment of Potential Ischaemic Heart Disease with CTCA (RAPID-CTCA) trial enrolled 1749 patients with suspected acute coronary syndrome with half of them ultimately being diagnosed with unstable angina or myocardial infarction. Both BEACON and RAPID-CTCA trials compared CTCA with standard of care. The CARdiovascular Magnetic rEsoNance imaging and computed Tomography Angiography (CARMENTA) trial randomly assigned 207 patients to either cardiac magnetic resonance imaging, CTCA, or standard of care in patients with suspected non-ST-segment elevation myocardial infarction who had cardiac troponin elevation but an inconclusive electrocardiogram.

### Invasive coronary angiography and coronary revascularisation

Reflecting the lower risk trial population, only a minority of patients in the BEACON trial underwent invasive coronary angiography (13–17%) or coronary revascularisation (7–9%), and the 30-day rates of these procedures were unaffected by CTCA.^46^ In the overall RAPID-CTCA trial population, the rate of invasive coronary angiography was lower with CTCA than with standard of care (54% vs  61%) although there was no difference in the rates of coronary revascularisation.^47^ For those with cardiac troponin elevation in the RAPID-CTCA trial, the findings remained consistent, suggesting CTCA allows for the better selection of patients for coronary revascularisation even in higher risk patients.^48^ In the CARMENTA trial, both cardiac magnetic resonance imaging and CTCA reduced the proportion of patients referred to invasive coronary angiography during index hospitalisation (87 and 66% vs  100% for standard of care, respectively) and at one year (88 and 70% vs  100%). In contrast, the diagnostic yield for the presence of diameter stenosis ≥70% was greater with CTCA than with cardiac magnetic resonance imaging or standard of care (85% vs 69 and 61%, respectively).^49^ Thus, these three trials demonstrate that CTCA reduces the requirement for invasive coronary angiography without affecting the use of coronary revascularisation especially in patients with suspected non-ST-segment elevation myocardial infarction. This has implications for resource management in hospitals where CTCA can help efficiently identify those who require invasive coronary angiography and access to coronary revascularisation.

### Non-invasive ischaemia testing and length of stay

There is always a concern that the introduction of new tests will lead to so-called test inflation where new or incidental findings will lead to further testing. Indeed, in low-risk and asymptomatic populations, this is an inherent concern. However, in general, CTCA was associated with a reduced need for downstream or layering of testing in patients with non-ST-segment elevation myocardial infarction. In keeping with the rates of invasive coronary angiography, CTCA was associated with a reduced requirement for downstream non-invasive ischaemia testing. This was seen across all three trials and resulted in similar lengths of stay. Overall, these trials would suggest that CTCA has no or minimal impact on length of stay but does reduce the need for downstream non-invasive ischaemia testing without impacting on overall healthcare costs (.

## Clinical outcomes of non-ST-segment elevation myocardial infarction

Early randomised controlled trials of CTCA recruited low-risk patients with acute chest pain who tended to be younger, with greater female sex representation and more cardiovascular risk factors (Table 2). They were evaluated with previous generations of less sensitive cardiac troponin assays, and overall rates of myocardial infarctions were very low at less than 1%.^18,19^ The more recent randomised controlled trials evaluating the effects of CTCA in the era of high-sensitivity cardiac troponin testing recruited much higher risk populations with rates of index non-ST-segment elevation myocardial infarction between 5 and 70%, and mean GRACE scores of approximately 115.

**Table 2.** Summary of background risk and clinical outcomes in major randomised controlled trials in suspected or diagnosed non-ST-segment elevation myocardial infarction

	Patients with low risk^*a*^					Patients with intermediate risk^*b*^		
	ACRIN PA 4005	ROMICAT II	CATCH	CT-COMPARE	CAD-Man	BEACON	RAPID-CTCA	CARMENTA
Age, yrs	49	54	56	52	60	54	62	64
Females, %	53	47	43	42	50	47	36	38
Diabetes mellitus, %	14	17	11	7	14	13	18	11
Hypertension, %	51	54	42	31	68	44	47	47
Hyperlipidaemia, %	27	45	38	25	53	35	40	34
Former or current smoker, %	33	49	64	23	53	44	61	38
Family history of CAD, %	29	27	25	33	12	42	31	46
Previous CAD, %	1	0	14	0	0	0	34	0
Ischaemic ECG, %	3	0	0	0	0	21	61	37
Elevated cardiac troponin, %	NR	0	0	0	NR	5	57	100
GRACE score	NR	NR	NR	NR	NR	83	115	115
GRACE > 140, %	NR	NR	NR	NR	NR	2	23	29
Downstream clinical events (CTCA *vs* control), %	1 *vs* 1 at 30 days	NR	11 *vs* 16 at median 19 months (including revascularisation and readmission for chest pain)	2 *vs* 1 at 1 year	4 *vs* 4 at median 3.3 years (including unstable angina, revascularisation, and stroke)	10 *vs* 9 at 30 days (including revascularisation)	6 *vs* 6 at 1 year	16 *vs* 23 at mean 1.3 years (including revascularisation, hospitalised heart failure, and procedure-related complications)
Overall long-term death or myocardial infarction during follow-up, %	NR	NR	1 at median 19 months	1 at 1 year	1 at median 3.3 years	NR	6 at 1 year	4 at median 1.3 years

CAD, coronary artery disease; CTCA, computed tomography coronary angiography; ECG, electrocardiogram; GRACE, Global Registry of Acute Coronary Events; NA, not applicable; NR, not reported.

The RAPID-CTCA trial reported the proportion of patients having abnormal ECG.

Clinical events were not consistently reported, and definitions varied between trials.

a Patients had neither history of CAD, ischaemic ECG, nor cardiac troponin elevation.

b Patients had either history of CAD, ischaemic ECG, or high-sensitivity cardiac troponin elevation.

Downstream clinical event rates are clearly determined by the length of follow-up and the risk of patients included in the trials. The follow-up duration of the BEACON trial was 30 days, and there was only one death and four possible recurrent acute coronary syndromes in 500 patients. The one-year event rate of death or myocardial infarction was comparable between the RAPID-CTCA (6%) and the CARMENTA (4%) trials. Nevertheless, patients with cardiac troponin elevation in the RAPID-CTCA trial appeared to have the highest risk as the one-year rate of death or myocardial infarction reached 8%.^48^ In the RAPID-CTCA and the CARMENTA trials, CTCA was associated with similar, albeit numerically lower, rates of major adverse cardiovascular events at one year: hazard ratio: 0.91 (95% confidence interval: 0.62–1.35) in the RAPID-CTCA trial and 0.64 (95% confidence interval: 0.18–2.27) in the CARMENTA trial.

Overall, contemporary trials enrolling higher risk patients including those with non-ST-segment elevation myocardial infarction have suggested that early CTCA can lead to accelerated management and tailored investigations and treatments but does not have a major impact on near- and short-term major adverse cardiovascular events. The high sensitivity and negative-predictive value of high-sensitivity cardiac troponin assays have meant that there is little scope for CTCA to identify unrecognised cases of non-ST-segment elevation myocardial infarction. Thus, there is no opportunity to prevent recurrent immediate or intermediate clinical events for those already diagnosed with non-ST-segment elevation myocardial infarction. However, CTCA does have a role in identifying those with cardiac troponin elevation who do not have Type 1 myocardial infarction and thereby improves on specificity for the diagnosis of non-ST-segment elevation myocardial infarction.

## Risk stratification of non-ST-segment elevation myocardial infarction

Initial risk stratification is essential in the clinical pathway for non-ST-segment elevation myocardial infarction in order to allocate appropriate therapies proportionate to the projected risk. Currently recommended risk stratification schemes using clinical variables offer good predictive value for near- and short-term outcomes.^5^ However, those clinical variables may not always represent a target that could be modified to improve outcomes, and the recommended risk scores and models do not always have good long-term discriminative performance.^50–52^ In contrast, cardiac imaging can potentially identify treatable targets, recommending treatment selection, as well as providing more powerful risk stratification.^53,54^

In patients with stable chest pain, CTCA-defined risk stratification is superior to functional testing for myocardial ischaemia,^55,56^ a finding that was recently reaffirmed in the International Study of Comparative Health Effectiveness with Medical and Invasive Approaches (ISCHEMIA) trial.^57^ For patients with suspected or diagnosed non-ST-segment elevation myocardial infarction, similar excellent risk prediction is seen with CTCA. In the VERDICT trial, the long-term (a median of 4.2 years) follow-up of 978 patients who underwent CTCA suggested that the presence of diameter stenosis ≥50% by CTCA was associated with an increased risk of major adverse cardiovascular events independent of cardiac troponin elevation.^58^ In addition, the presence of diameter stenosis ≥50% in left main coronary artery, proximal left anterior descending artery, or two or more vascular territories by CTCA identified patients at the highest risk of long-term adverse outcomes. Finally, among those without diameter stenosis ≥50% by CTCA, the subsequent findings of invasive coronary angiography did not further distinguish patients at increased risk.

## Future directions

### Diagnosis

**Figure 2.**
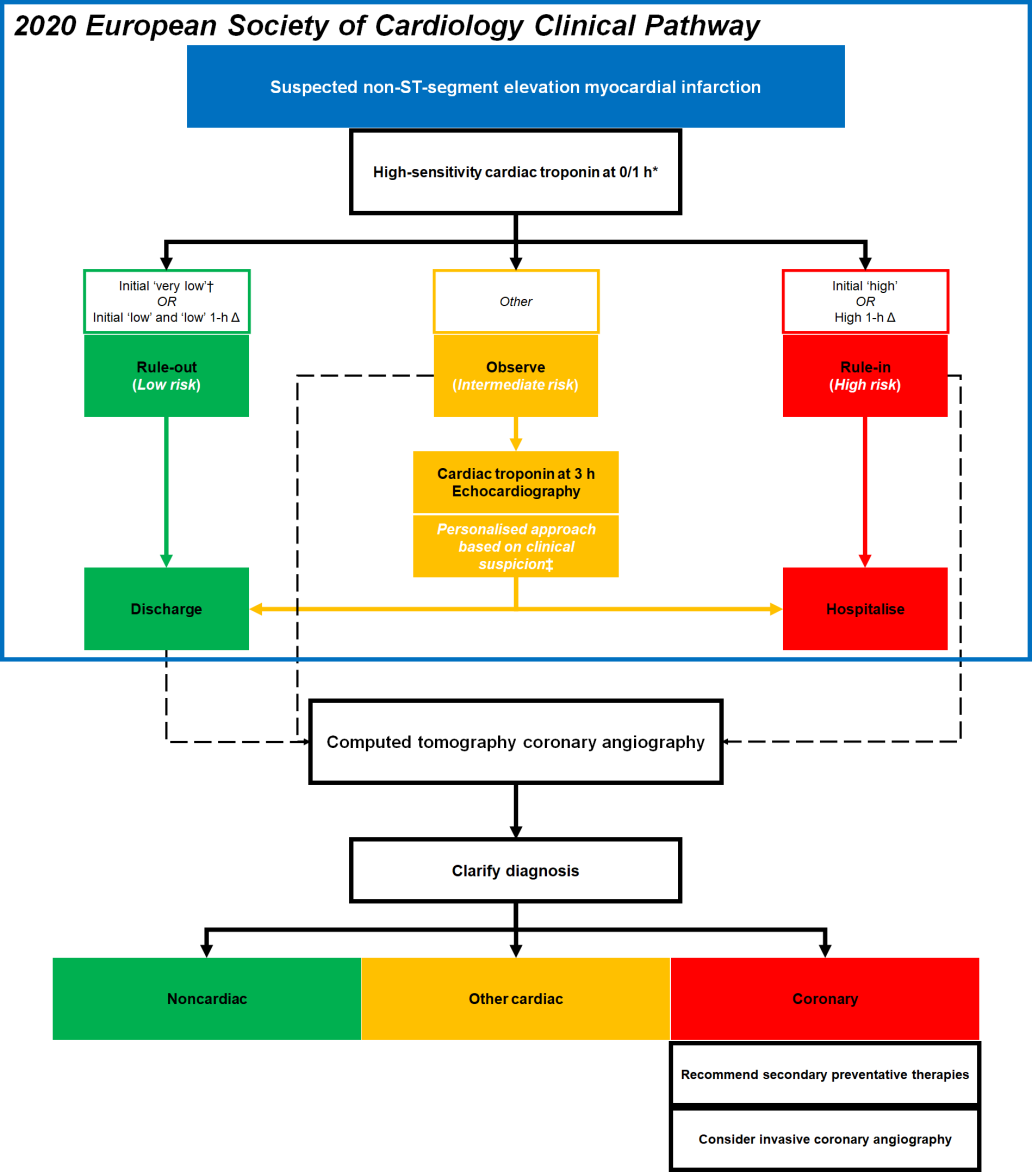
The schematic diagram proposing the application of computed tomography coronary angiography in the clinical pathway in conjunction with the European Society of Cardiology 0/1 h rule-in and rule-out algorithm.

Current diagnostic algorithms have accelerated the management of most patients with acute chest pain by excluding myocardial infarction. However, it is still uncertain and challenging in those assigned to the observe zone of cardiac troponin where a personalised approach based on clinical suspicion is required (Figure 2).^21^ On the other hand, most cardiac troponin elevations are related to either Type 2 myocardial infarction or myocardial injury. Again, the differentiation between Type 1 myocardial infarction, Type 2 myocardial infarction, and myocardial injury can be challenging, and the optimal investigations and treatments for these conditions remain to be established. This has led to the design of several clinical studies investigating the use of CTCA in patients with suspected or diagnosed non-ST-segment elevation myocardial infarction (Table 3).

**Table 3.** Summary of ongoing clinical studies of computed tomography-based assessment in suspected or diagnosed non-ST-segment elevation myocardial infarction

	ClinicalTrials.gov Identifier	Study design	Country	Patient no	Key inclusion	Key exclusion	Studied treatment	Primary endpoint
Diagnosis	NCT02828761	Diagnostic, randomised controlled trial	United States	800	Acute chest pain at intermediate risk by HEART score	Previous CAD A cardiac troponin concentration greater than three times the laboratory threshold	Coronary calcium scoring *vs* SoC	Major adverse cardiovascular events at 30 days
	NCT03129659 COURSE	Diagnostic, single group assignment	Netherlands	230	Suspected acute coronary syndrome in the observe zone based on high-sensitivity cardiac troponin	Previous CAD	CTCA	Diagnostic accuracy to identify acute coronary syndrome
	NCT03583320 PROTECT	Diagnostic, randomised controlled trial	United Kingdom	250	Suspected acute coronary syndrome with initial high-sensitivity cardiac troponin in the intermediate range	Previous CAD	CTCA *vs* SoC	Length of stay up to 1 year
	NCT04864119 DEFINE TYPE 2 MI	Observational	United States	50	Type 2 myocardial infarction	Other types of myocardial infarction	FFRct	Prevalence of obstructive CAD
Long-term outcomes	NCT04748237 FAST-CCTA	Diagnostic, randomised controlled trial	Sweden	3500	Suspected acute coronary syndrome with an intermediate risk (HEART score >3)	Myocardial infarction Previous CAD	CTCA *vs* SoC	Death, readmission because of myocardial infarction or unstable angina requiring revascularisation at 3 years
	NCT03952351 TARGET-CTCA	Screening, randomised controlled trial	United Kingdom	2270	Suspected acute coronary syndrome and a high-sensitivity cardiac troponin concentration between 5 ng/L and the 99th centile upper reference limit	Myocardial infarction	CTCA *vs* SoC	Cardiac death or myocardial infarction at median of 3 years

CAD, coronary artery disease; CTCA, computed tomography coronary angiography; FFRct, Fractional flow reserve by computed tomography; HEART, History, ECG, Age, Risk factors, and Troponin; SoC, standard of care.

The atherosclerotic plaque burden measured by calcium score is a simple gauge for long-term cardiovascular outcomes independent of diameter stenosis,^59^ and coronary calcium scoring can be a useful gatekeeper for deferral of invasive coronary angiography in patients with acute chest pain.^60^ The Randomized Controlled Trial of Early Coronary Calcium Scoring and Standard Care in Emergency Department Chest Pain Patients (ClinicalTrials.gov identifier, NCT02828761) is currently recruiting patients with intermediate risk and a cardiac troponin concentration within three times upper reference limit to evaluate the effect of coronary calcium scoring on diagnosis and management for patients in the observe zone. Another two studies, the Coronary CT Angiography for Improved Assessment of Suspected Acute Coronary Syndrome With Inconclusive Diagnostic Work-up (COURSE; ClinicalTrials.gov identifier, NCT03129659) and the Prospective RandOmised Trial of Emergency Cardiac CT (PROTECT; ClinicalTrials.gov identifier, NCT03583320) will throw light on the effectiveness of CTCA in patients with a ‘non-diagnostic’ cardiac troponin concentration under the current clinical pathway.

**Figure 3.**
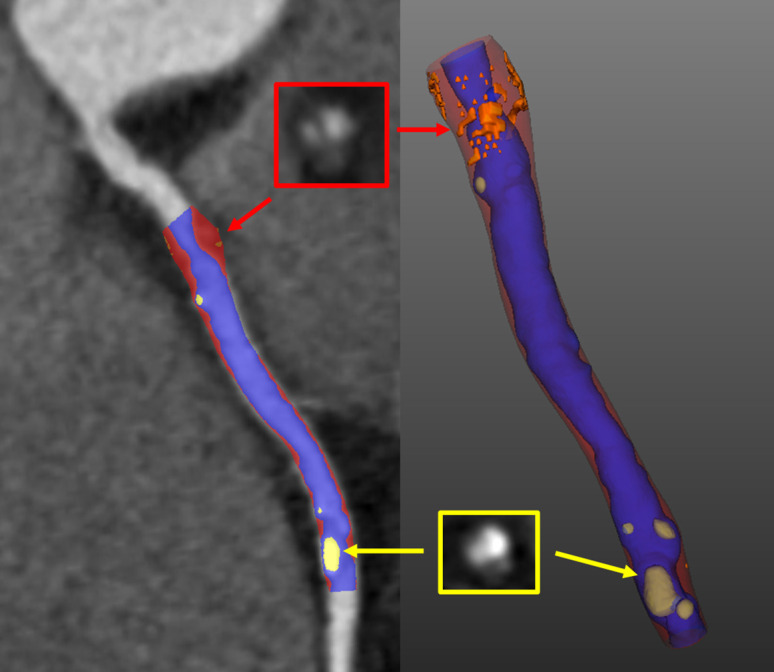
Computed tomography coronary angiography plaque analysis

CTCA visualises the coronary artery wall, enabling characterisation of atherosclerotic plaque morphology. Indeed, several visually assessed adverse plaque characteristics have been described, including positive remodelling, low attenuation, spotty calcification, and the napkin-ring sign. In patients with stable chest pain, these adverse features predict major adverse cardiovascular events.^61,62^ Beyond qualitative assessment, semi-automated quantification allows for a reproducible and detailed breakdown of atherosclerotic plaque morphology (Figure 3).^63,64^ It has been extensively validated against intracoronary imaging.^65^ Low-attenuation plaque is of particular interest since it correlates with the lipid-rich necrotic core central to the pathogenesis of Type 1 myocardial infarction. In addition, the burden of low-attenuation plaque is associated with an increase in cardiovascular risk score, calcium score, coronary artery diameter stenosis, and the incidence of myocardial infarction.^66^ In fact, compared with asymptomatic patients, those with acute chest pain have a greater volume of total and low-attenuation plaque, particularly those with non-ST-segment elevation myocardial infarction.^67^ In the RAPID-CTCA trial, the burden of total, noncalcified, and low-attenuation plaque were the strongest predictors of death or myocardial infarction at one year, independent of GRACE score and the presence of diameter stenosis ≥70%, among patients with suspected acute coronary syndrome.^68^ Furthermore, CTCA along with quantitative plaque analysis potentially helps discriminate acute coronary syndrome by characterising adverse plaque features.^69,70^

Additional computational fluid dynamics and advanced molecular imaging may enhance the identification of adverse features that are culprit for incident acute coronary syndrome.^71,72^ The Determining the Mechanism of Myocardial Injury and Role of Coronary Disease in Type 2 Myocardial Infarction (DEMAND-MI) study showed that two-thirds of patients with Type 2 myocardial infarction had coronary artery disease of any severity, of which half had not been identified previously. In addition, many of them were not treated with preventative therapies, suggesting missed treatment opportunities.^25^ Further substudies of the DEMAND-MI study and the DEFINing the PrEvalence and Characteristics of Coronary Artery Disease Among Patients With TYPE 2 Myocardial Infarction Using CT-FFR (DEFINE Type 2 MI; ClinicalTrials.gov identifier, NCT04864119) study will use CTCA-based techniques to explore the prevalence of coronary artery disease, haemodynamically significant stenosis, and plaque characteristics among patients with Type 2 myocardial infarction. This information will increase our understanding of pathogenesis of Type 2 myocardial infarction and suggest potential treatment opportunities.

### Ischaemia assessment and coronary revascularisation

Although CTCA is excellent at identifying normal coronary arteries and avoiding unnecessary invasive coronary angiography,^47,49^ the specificity of CTCA in discriminating anatomical (≥50% diameter stenosis) or functional (fractional flow reserve ≤0.80) obstruction remains imperfect.^73^ Fractional flow reserve derived from CTCA is a promising approach that improves discrimination of those at higher risk for future events.^74^ However, its use has not resulted in further improvement of major adverse cardiovascular events in patients with stable chest pain.^75^ The CArdiac cT in the treatment of acute CHest pain-2 (CATCH-2) trial showed that additional computed tomography perfusion imaging further reduced the utilisation of invasive coronary angiography in low-risk patients with acute chest pain,^76^ but it was underpowered to evaluate clinical outcomes. These findings are not definitive, and the role of CTCA-derived fractional flow reserve or computed tomography myocardial perfusion remains to be established in non-ST-segment elevation myocardial infarction. Ultimately, any advanced computed tomography-based functional assessment will need to demonstrate incremental diagnostic and prognostic value over CTCA in the value-based health care.^77^

### Long-term outcomes

The principal advantage of CTCA is to identify unrecognised coronary atherosclerosis regardless of stenosis severity as an opportunity to apply preventative interventions, such as antiplatelet and statin therapies. The benefit of such interventions take many years to accrue as demonstrated in patients with stable chest pain.^17,78^ Thus, the greatest opportunity for CTCA to improve long-term clinical outcomes in those with acute chest pain may be in those who have myocardial infarction excluded but may still be at a high risk of future long-term cardiovascular events. Two ongoing randomised controlled trials, the Randomized Evaluation of Coronary Computed Tomographic Angiography in Intermediate-risk Patients Presenting to the Emergency Department With Chest Pain (FAST-CCTA; ClinicalTrials.gov identifier, NCT04748237) and the Troponin in Acute Chest Pain to Risk Stratify and Guide EffecTive Use of Computed Tomography Coronary Angiography (TARGET-CTCA; ClinicalTrials.gov identifier, NCT03952351) trials, will determine the effectiveness of CTCA on long-term (≥3 years) major adverse cardiovascular events in such patients.

## Conclusions

In patients with suspected non-ST-segment elevation myocardial infarction, electrocardiography and high-sensitivity cardiac troponin testing will remain the initial step for clinical evaluation, and invasive coronary angiography is still the routine clinical tool to determine the strategy of coronary revascularisation. CTCA has an increasingly important role in avoiding unnecessary invasive coronary angiography and reducing downstream non-invasive functional testing for myocardial ischaemia especially among those with low or intermediate risk. Moreover, CTCA is an excellent gatekeeper for the cardiac catheterisation laboratory, provides complementary information for patients with myocardial infarction in the absence of obstructive coronary artery disease, and can highlight alternative or incidental diagnoses for patients with non-ST-segment elevation myocardial infarction. There are several ongoing studies evaluating CTCA and its associated technologies that will define and potentially expand its application in patients with suspected or diagnosed non-ST-segment elevation myocardial infarction.
